# Effects of health insurance on patient demand for physician services

**DOI:** 10.1186/s13561-020-00291-y

**Published:** 2020-09-17

**Authors:** Jerome Dugan

**Affiliations:** Department of Health Services, School of Public Health, University of Washington, 1959 NE Pacific St Magnuson Health Sciences Center, Room H-680, Box 357660, Seattle, WA 98195-7660 USA

**Keywords:** Healthcare management, Chronic disease, Health insurance

## Abstract

**Background:**

In recent years, policymakers have sought to reduce health disparities between the insured and uninsured through a federal health insurance expansion policy; however, disparities continue to persist among the insured population. One potential explanation is that the use of healthcare services varies by the type of health insurance coverage due to differences in the design of coverage. The aim of this study is to examine whether health insurance coverage type is associated with the structure and use of healthcare services.

**Methods:**

The nationally representative Medical Expenditure Panel Survey and multinomial logistic regression are used to estimate the effects of different types of health coverage on the combinations of routine and emergency care sought and received.

**Results:**

The multinomial logistic regression analysis for the overall sample revealed privately insured respondents reported higher use of routine care only (24.33%; *p* < 0.001) and lower use of emergency room care only (− 2.13%; *p* < 0.01) than the uninsured. The publicly insured reported similar trends for use of routine care only (17.93%; *p* < 0.001) as the privately insured, as compared to the uninsured. Both the privately and publicly insured reported higher use of a mixture of care; however, publicly insured were more likely to use a mixture of care (8.57%, *p* < 0.001).

**Conclusion:**

The results show that health insurance is associated with higher use of the physician services, but does not promote the use of cost-effective schedules of care among the publicly insured.

## Introduction

The Social Security Act Amendments (Pub L N. 89–97), passed in 1965, were designed to address the low rates of insurance among financially vulnerable households not receiving coverage from an employer by creating the Medicare insurance program for older adults over 65 and the Medicaid insurance program for low income mothers and children. However, in later years, coverage expansions have widened to include the remainder of the uninsured population using both a public and private mechanism. More specifically, the Affordable Care Act (Pub L N. 111–148) reduced the uninsured rate across the population by allowing states to expand Medicaid to include low-income adults, providing premium subsidies to help improve the affordability of private health plans, and imposing an individual mandate requiring all persons not receiving government or employer-based insurance to retain coverage. The most recent expansion policies under the ACA were able to lower the uninsured rate among non-elderly population from 46.5 million to 27.4 million between 2010 and 2017, thus highlighting the strength of using either a public or private insurance as a mechanism to address the uninsured rate and number of uninsured.

In the next round of reforms, policymakers are debating whether to move forward with a public takeover of the U.S. healthcare insurance system or alternatively push for a tiered set of private healthcare plans to cover the remainder of the uninsured population. Researchers in the US have documented that insurance status plays an important role in individual health, as the uninsured are the least likely to use preventative medical services, are the most likely to encounter financial barriers to access care, and report the highest rates of preventable hospitalizations [1–7]; however, less is known about how use of care varies by health insurance coverage type (e.g., no insurance, public insurance, private insurance). This paper aims to examine how the structure and scope of medical services sought and received vary by insurance status.

## Methods

### Sources of data

The Medical Expenditure Panel Survey (MEPS) provides the most current and comprehensive individual-level data for evaluating medical consumption, expenditure, and health status. This study uses data from the household component of the MEPS, which collects data from a nationally representative subsample of the National Health Interview Survey participants from the previous year. The base year for the study is 2015, the year following the implementation of the ACA’s core health insurance expansion provisions, and the final year is 2017, the most recent year that comprehensive data are available. The analysis dataset was restricted to respondents aged 26 to 64 to avoid potential cofounding effects of the young adult health insurance coverage provisions (under age 26) and Medicare (over the age 64).

### Outcome measures

Medical treatment bundles – a group of individual medical services that are used together to manage personal health – are utilized to capture patients’ tastes for different patterns of healthcare utilization. In this study, several measures of individual utilization were combined into a single categorical measure of medical treatment. The following three treatment bundles were considered: two or more routine office-based visits only, two or more emergency room visits only, and any mixture of two or more routine or emergency room visits. Respondents also have the option to make one or less medical visits of either kind. It is important to note that medical treatment bundles used in this study do not represent an absolute measure of utilization, but rather, are designed to reveal patterns of utilization.

### Control variables

For our sample of adults aged 26 to 64, respondents were identified as being publicly insured if they were recipients of Medicaid (the state-federal insurance program for low-income and disabled persons or some other state-federally sponsored insurance plan) TRICARE (the health care program of the United States Department of Defense Military Health System), or other public hospital/physician programs. To avoid the potential confounding effects of Social Security Insurance (SSI) and Social Insurance Disability (SSD), programs that provide both cash assistance and Medicare to individuals with a qualified disability status, Medicare beneficiaries are excluded. Respondents were identified as being privately insured if they obtained their coverage from their employer or a private health insurance marketplace. Respondents who report no private or public insurance coverage were identified as being uninsured. In addition to controlling for health insurance coverage type, a number of socioeconomic variables to reduce confounding bias are included. Socioeconomic control variables included age, sex, race/ethnicity, educational attainment, personal income. Census region and survey year were also included in the regression analysis to control for geographic and time effects.

### Statistical analysis

This study utilizes a discrete choice framework, where the choice set is defined by set of medical treatment bundles and the decision makers are patients with different health insurance coverage statuses [8–10]. Conditioned on a coverage type, a patient’s optimization problem is to select the medical treatment bundle that minimizes their risk of an acute event subject to the constraints imposed by their individual health insurance status. Empirical estimation for this optimization problem is performed using multinomial logistic regression (MLR) [10, 11], which models the probability that patient will choose a given medical treatment bundle over an alternative bundle. The MLR coefficients have been converted from odds ratios into marginal effects to improve the interpretability of results. All regressions are weighted to adjust for oversampling and include robust standard errors to address heteroskedastic bias. The theoretical and empirical model is described in more detail in Appendix A.

## Results

### Summary statistics

Population-weighted socioeconomic characteristics of respondents aged 26 to 64 for the overall study population are described in Table 1, Column 1. For the overall sample (*n* = 47,252), the mean age was 44.46 years, and 51.21% of the population were men. Respondents with private insurance represent 77.44% of the sample, respondents with public insurance represent 11.34% of the sample, and uninsured respondents represent 11.21% of the sample. All major racial groups are well represented in the sample, with White non-Hispanics, Black non-Hispanics, and Hispanics representing 61.37, 11.93, and 17.34%, respectively. High school dropouts represent 10.11% of the sample, while respondents with a high school degree or at least some college experience represent 26.59 and 63.30%, respectively.

**Table 1:** Descriptive Statistics of Adults by Medical Treatment Bundle

Characteristics	All	Routine Visits Only ^a^	Emergency Visits Only ^b^	Mixture of Visits ^c^	One or Less Visits ^d^	*χ*^2^*P*
	(1)	(2)	(3)	(4)	(4)	(5)
Unweighted sample size, *N*	47,252	22,454	1378	4530	18,890	
Mean age, y	44.46 (11.26)	46.21 (11.21)	40.92 (10.45)	45.59 (11.39)	41.84 (10.80)	< 0.001
Gender						
Female, %	48.79 (49.99)	42.69 (49.46)	53.50 (49.90)	37.41 (48.39)	60.36 (48.92)	< 0.001
Male, %	51.21 (49.99)	57.31 (49.46)	46.50 (49.90)	62.59 (48.39)	39.64 (48.92)	< 0.001
Geographic Region						
Northeast, %	17.66 (38.14)	18.78 (39.06)	15.44 (36.14)	18.34 (38.70)	16.00 (36.66)	< 0.001
Midwest, %	20.81 (40.60)	21.01 (40.74)	22.23 (41.60)	23.29 (42.27)	19.78 (39.84)	< 0.001
South, %	37.34 (48.37)	36.33 (48.10)	42.70 (49.48)	38.09 (48.57)	38.26 (48.60)	< 0.001
West, %	24.18 (42.82)	23.87 (42.63)	19.63 (39.74)	20.28 (40.21)	25.96 (43.84)	< 0.001
Insurance Coverage						
Private insurance, %	77.44 (41.80)	84.96 (35.74)	54.88 (49.78)	69.83 (45.90)	69.96 (45.85)	< 0.001
Public insurance, %	11.34 (31.71)	9.59 (29.44)	24.37 (42.95)	23.25 (4224)	9.93 (29.91)	< 0.001
Uninsured, %	11.21 (31.56)	5.45 (22.70)	20.75 (40.57)	6.922 (25.39)	20.11 (40.09)	< 0.001
Race/Ethnicity						
White non-Hispanic, %	61.37 (48.69)	66.11 (47.34)	52.24 (49.97)	62.16 (48.50)	54.86 (49.76)	< 0.001
Black non-Hispanic, %	11.93 (32.41)	10.43 (30.56)	20.32 (40.25)	15.72 (36.40)	12.57 (33.15)	< 0.001
Other non-Hispanic, %	9.36 (29.12)	9.15 (28.82)	8.17 (27.39)	6.89 (25.33)	10.39 (30.51)	< 0.001
Hispanic, %	17.34 (37.86)	14.31 (35.03)	19.27 (39.46)	15.24 (35.94)	22.18 (41.55)	< 0.001
Educational Attainment						
No degree	10.11 (30.15)	7.52 (26.38)	17.61 (38.11)	12.20 (32.73)	12.86 (33.48)	< 0.001
High school degree/GED	26.59 (44.18)	23.38 (42.33)	33.69 (47.29)	28.49 (45.14)	30.31 (45.96)	< 0.001
At least some college	63.30 (48.20)	69.09 (46.21)	48.70 (50.01)	59.31 (49.13)	56.83 (49.53)	< 0.001

In addition to reporting personal characteristics for the overall sample, Table 1 also reports socioeconomic characteristics of respondents who make two or more routine visits only (Table 1, Column 2), two or more emergency visits only (Table 1, Column 3), a mixture of two or more routine and emergency visits (Table 1, Column 4), or one or less medical visits of either type (Table 1, Column 5). Medical utilization varied considerably across different medical treatment bundles, with 22,454 making routine visits only, 1378 making emergency visits only, 4530 making a mixture of visits, and 18,890 making one or less visits. Women are more likely than men to make emergency visits only (53.50%) or one or less visits (60.36%), while men are more likely than women to make routine visits only (57.71%) and mixture of visits (62.59%). White respondents, privately insured respondents, and respondents with at least some college are more likely to make physician visits of any type over all other insurance status, racial/ethnic, and educational attainment groups.

### Effects of health insurance coverage type of health care utilization

The multinomial logistic regression results summarizing the impact of insurance type on a patient’s use of office-based visits only, emergency room visits only, and a mixture of visits, as compared to inadequate utilization are reported in Table 2. The results of the overall sample (Panel A) show that adults with private insurance had a higher probability of utilizing routine office-based care (24.33%; *p* < 0.01), a lower probability of using emergency care only (− 2.13%; *p* < 0.01), and a high probability to use a mixture of routine and emergency care (1.27%; *p* < 0.10) than the uninsured. Similarly, the overall sample results show that adults with public insurance had a higher probability of utilizing routine care only (17.93%; *p* < 0.01) and a mixture of routine and emergency care (8.57%; *p* < 0.01) than the uninsured.

**Table 2:** Medical Treatment Regression Analysis, Multinomial Logistic Regression Analysis

	Routine Visits Only ^a^	Emergency Visits Only ^b^	Mixture of Visits ^c^	Routine Visits Only ^a^	Emergency Visits Only ^b^	Mixture of Visits ^c^
*Coefficient*	(1)	(2)	(3)	(4)	(5)	(6)
	A. Overall Sample (*N* = 36,424)			B. White non-Hispanic (*N* = 15,407)		
Private insurance	24.33*** (1.09)	−2.13*** (0.26)	1.27* (0.74)	27.45*** (1.93)	− 2.02*** (0.37)	0.10 (1.15)
Public insurance	17.93*** (1.30)	0.25 (0.26)	8.57*** (0.76)	19.07*** (2.34)	0.09 (0.39)	7.19*** (1.25)
Uninsured	1	1	1	1	1	1
	C. Black non-Hispanic (*N* = 6315)			D. Hispanic (*N* = 10,773)		
Private insurance	26.36*** (2.44)	−4.90*** (0.69)	2.05 (1.71)	19.46*** (1.45)	−1.46*** (0.46)	2.71** (1.13)
Public insurance	23.13*** (2.76)	−2.00*** (0.72)	10.10*** (1.77)	13.62 (1.72)	1.44*** (0.48)	9.57*** (1.12)
Uninsured	1	1	1	1	1	1

*Notes:* The multinomial logistic regression coefficients have been converted into marginal effects to improve interpretability. Standard deviations are reported in parentheses. All regressions include individual controls and time trends. The reference category is uninsured respondents

^a^ Routine visits only, 2+ routine office based visits only

^b^ Emergency visits only, 2+ emergency room visits only

^c^ Mixture of visits, 2+ routine and emergency room only

*Significant at 10% confidence level

**Significant at 5% confidence level

***Significant at 1% confidence level

In order to understand how the effects of insurance coverage on utilization varies across race/ethnicity, MLR models were also estimated for the three largest racial/ethnic groups: White non-Hispanics (Table 1, Panel B), Black non-Hispanics (Table 1, Panel C), and Hispanics (Table 1, Panel C). The racial/ethnic subgroup analysis revealed White non-Hispanics reported similar trends of the overall study population. While Black non-Hispanics and Hispanics also report similar trends as the overall population, Black non-Hispanic respondents with public insurance reported lower use of emergency visits only (2.00%; *p* < 0.01) and publicly insured Hispanics reported a higher use of emergency visits only (1.44%; *p* < 0.01) than uninsured Black non-Hispanics and Hispanics, respectively.

### Patterns of healthcare utilization by disease group

Table 3 also presents the medical treatment regression results for individual diseases, focusing on hypertension respondents (Table 3, Panel A), coronary heart disease and stroke (CHDS) respondents (Table 3, Panel B), diabetes respondents (Table 3, Panel C), and cancer respondents (Table 3, Panel D). The purpose of the chronic disease subgroup analysis is to examine the role disease status plays in physician use. Respondents were identified as having hypertension, CHDS, diabetes, or cancer. Respondents were identified as having CHDS if they were ever told by a doctor or other health professional that they had coronary heart disease, a heart attack, or stroke. CHDS patients are organized into a single diagnosis group because both groups share the same pathophysiology [12–14].

**Table 3:** Medical Treatment Regression Analysis, Multinomial Logistic Regression Analysis

	Routine Visits Only ^a^	Emergency Visits Only ^b^	Mixture of Visits ^c^	Routine Visits Only ^a^	Emergency Visits Only ^b^	Mixture of Visits ^c^
*Coefficient*	(1)	(2)	(3)	(4)	(5)	(6)
	A. Hypertension (*N* = 10,425)			B. CHDS (*N* = 3759)		
Private insurance	18.68*** (1.52)	−3.09*** (0.45)	0.68 (1.25)	16.04*** (2.73)	−3.80*** (0.85)	1.33 (2.44)
Public insurance	10.66*** (1.75)	−0.88** (0.44)	12.86*** (1.27)	5.92** (3.00)	− 1.40* (0.74)	16.04*** (2.47)
Uninsured	1	1	1	1	1	1
	C. Diabetes Mellitus (*N* = 2945)			*D. cancer* (*N* = 2013)		
Private insurance	11.18*** (2.76)	−1.83** (0.77)	−2.02 (2.46)	14.19 *** (4.20)	− 2.33*** (0.86)	−0.69 (3.70)
Public insurance	−0.07 (3.04)	−0.70 (0.75)	13.92*** (2.47)	5.29 (4.68)	− 1.85** (0.93)	11.96*** (3.85)
Uninsured	1	1	1	1	1	1

*Notes:* The multinomial logistic regression coefficients have been converted into marginal effects to improve interpretability. Standard deviations are reported in parentheses. All regressions include individual controls and time trends. The reference category is uninsured respondents. CHDS, coronary heart disease and stroke

^a^ Routine visits only, 2+ routine office based visits only

^b^ Emergency visits only, 2+ emergency room visits only

^c^ Mixture of visits, 2+ routine and emergency room only

*Significant at 10% confidence level

**Significant at 5% confidence level

***Significant at 1% confidence level

The results of the hypertension group (Table 3, Panel A) show that adults with private insurance had a higher probability of utilizing routine office-based care (18.68%; *p* < 0.001) and a lower probability of using emergency care only (− 3.09%; *p* < 0.001) than the uninsured. Private insurance was not associated with an increased propensity of using a mixture of routine and emergency room care. Similarly, adults with public insurance had a higher probability of utilizing routine care only (10.66%; *p* < 0.001) and less likely to utilize emergency care only (− 0.88%; *p* = 0.096) than the uninsured. Although adults with private and public insurance are more likely to use a mixture of routine and emergency care than the uninsured, patients with public insurance reported a substantially higher probability to use a mixture of routine and emergency care (18.41%; *p* < 0.001) than the uninsured. The results for the CHDS, diabetes, and cancer groups are consistent with the hypertension group results in sign and magnitude.

### Alternative specifications

In the theoretical framework of this paper (Appendix A), it was argued that if an individual does not consume a treatment bundle in J, then their post-treatment health state is equal to their pre-treatment health state [15]. Because the emergency room setting alone is not the appropriate place for all healthcare needs (e.g., prescription filling), it may be unlikely that emergency only is capable of helping maintain health status. To address this concern, the main analysis is re-estimated without an emergency only medical treatment bundle (Table 4, Panel A). The regression results from this alternative model are similar in sign and magnitude as the regression results of the main model. Second, following the passage of the ACA, income played an important role in determining the amount of government support individual households received to access health insurance: households making less than 133% FPL are eligible for Medicaid, households making between 133 and 400% FPL qualify for a premium tax credit, and households making over 400% FPL receive no ACA related support. To examine the differential impact of government support, the analysis is re-estimated for three income categories (Table 4, Panels B, C, D). The coefficient estimates from the income analysis are consentient with the trends of the overall population.

**Table 4:** Alternative Specifications, Multinomial Logistic Regression Analysis

	Routine Visits Only ^a^	Emergency Visits Only ^b^	Mixture of Visits ^c^	Routine Visits Only ^a^	Emergency Visits Only ^b^	Mixture of Visits ^c^
*Coefficient*	(1)	(2)	(3)	(4)	(5)	(6)
	A. Emergency Visits Only Added to Other Category (*N* = 36,424)			B. ≤133% FPL (*N* = 7707)		
Private insurance	2.43*** (1.1)		1.3* (0.7)	21.2*** (2.1)	−4.2*** (1.1)	3.2* (1.9)
Public insurance	1.73*** (1.3)		8.5*** (0.8)	19.4*** (1.9)	−1.1 (0.7)	12.5*** (1.6)
Uninsured	1	1	1	1	1	1
	C. 133 to 400% FPL (*N* = 15,466)			C. > 400% FPL (*N* = 13,251)		
Private insurance	21.8*** (1.5)	−1.3*** (0.4)	0.025** (1.1)	0.205*** (0.026)	−1.3*** (0.3)	2.5 (1.6)
Public insurance	14.2*** (2.0)	0.9* (0.5)	8.3*** (1.2)	16.9*** (4.4)	−0.5 (0.7)	4.0* (2.2)
Uninsured	1	1	1	1	1	1

*Notes:* The multinomial logistic regression coefficients have been converted into marginal effects to improve interpretability. Standard deviations are reported in parentheses. All regressions include individual controls and time trends. The reference category is uninsured respondents. FPL, federal poverty level

^a^ Routine visits only, 2+ routine office based visits only

^b^ Emergency visits only, 2+ emergency room visits only

^c^ Mixture of visits, 2+ routine and emergency room only

*Significant at 10% confidence level

**Significant at 5% confidence level

***Significant at 1% confidence level

### Addressing endogeneity and omitted variables bias

A major assumption of the analysis is that coverage status is not endogenously related to use of medical treatment bundles; however, this assumption could be violated if persons select their coverage in anticipation of future healthcare needs. Furthermore, the construction of public and private insurance as uniform health insurance plan types ignores the potential role of non-price rationing across plan types and the mechanism of enrollment (i.e., adverse selection) into health insurance plans can confound any estimates of the effects of coverage on medical treatment. To investigate these issues, the exogenous variation in the timing of acute health events and an age-based eligibility threshold are utilized to address potential endogeneity. Furthermore, omitted variables bias is addressed by controlling for additional coverage characteristics of health insurance plans.

First, the main results are re-estimated using a sample limited to respondents who experienced an acute event/diagnosis (e.g., stroke, heart attack, cancer) within 1 year of being surveyed (Table 5, Panel A). The results are robust to this sampling restriction. Second, following [13], an RD model that exploits a sharp increase in coverage resulting from older adults becoming eligible for Medicare at age 65 is estimated (Table 5, Panel B). The results of the RD analysis are consistent with the main results, where public insurance respondents are more likely than the uninsured to make routine visits only. Third, additional attributes to public and private insurance are considered by incorporating managed care interaction effects for both public and private plans (Table 5, Panel C). The results of managed care analysis revealed no statistically significant managed care interaction effects.

**Table 5:** Addressing Endogeneity and Omitted Variables Bias, Multinomial Logistic Regression Analysis

	Routine Visits Only ^a^	Emergency Visits Only ^b^	Mixture of Visits ^c^	Routine Visits Only ^a^	Emergency Visits Only ^b^	Mixture of Visits ^c^
*Coefficient*	(1)	(2)	(3)	(4)	(5)	(6)
	A. Acute Event Occurred Within One Year of the Survey (*N* = 1274)			B. Regression Discontinuity at Medicare Eligibility Threshold (*N* = 2203)		
Private insurance	12.41** (6.03)	−3.98*** (1.45)	−1.00 (5.63)			
Public insurance	2.66 (6.31)	−2.85** (1.46)	13.76*** (5.51)			
RD Term (≥ 65)				37.96* (20.73)	−61.43** (27.61)	6.44 (7.52)
	C. Managed Care Effects (*N* = 36,424)			D. Detailed Coverage Type (*N* = 36,424)		
Private insurance	24.24*** (1.13)	−2.16*** (0.29)	1.33* (0.75)			
Public insurance	17.03*** (1.50)	0.20 (0.30)	8.17*** (0.81)			
Private insurance × managed care	0.28 (0.80)	0.11 (0.29)	−0.20 (0.48)			
Public insurance × managed care	2.20 (1.64)	0.12 (0.35)	0.96 (0.69)			
Job-Based Group Insurance				22.22*** (0.94)	−2.16*** (0.26)	−0.24 (0.55)
Other Group Insurance				17.24*** (4.55)	−1.97 (2.38)	−1.76 (2.55)
Individual (Non-Group)				14.17*** (1.21)	−1.48 (0.33)	0.91 (0.67)
Medicaid				12.56*** (1.08)	0.48** (0.24)	7.89*** (0.54)
Tricare/CHAMPVA				1.01 (2.22)	1.05* (0.60)	2.58** (1.22)
Other Public				14.49*** (5.21)	−5.26** (2.39)	−0.30 (2.49)
Uninsured	1	1	1	1	1	1

*Notes:* The multinomial logistic regression coefficients have been converted into marginal effects to improve interpretability. Standard deviations are reported in parentheses. All regressions include individual controls and time trends. The reference category is uninsured respondents

^a^ Routine visits only, 2+ routine office based visits only

^b^ Emergency visits only, 2+ emergency room visits only

^c^ Mixture of visits, 2+ routine and emergency room only

*Significant at 10% confidence level

**Significant at 5% confidence level

***Significant at 1% confidence level

As health insurance plans vary significantly within public and private insurance groups, the public/private insurance groups are decomposed into specific plan types (Table 5, Panel D). Three types of public, non-Medicare insurance plans (i.e., Medicaid, TRICARE/CHAMPVA, other public insurance) and three types of private insurance plans (i.e., job-based group insurance, other group insurance, non-group insurance) are examined in the health insurance attribute analysis. Regarding routine visits only, with an exception to TRICARE/CHAMPVA, all other insurance types reported a higher propensity to make routine visits only as compared to the uninsured. Regarding emergency visits only, among the privately insured, only persons with job-based group insurance reported a lower propensity to make emergency visits only (− 2.16%; *p* < 0.01). Among the publicly insured, Medicaid and TRICARE were more likely to make emergency room visits only at (0.48%; *p* < 0.05) and (1.05%; *p* < 0.10), respectively. Persons with other public insurance reported a lower propensity to make emergency visits only (− 5.26%; *p* < 0.05). Pubic insurance beneficiaries of any type reported no differences in mixtures of visits; however, Medicaid and TRICARE beneficiaries reported a higher propensity to make a mixture of visits at (7.89%; *p* < 0.01) and (2.58%; *p* < 0.05), respectively. The robust findings across all alternative specifications suggest that coverage status is not endogenous to medical treatment bundle choice.

## Discussion

The medical treatment analysis revealed two important results. First, the main results show that the uninsured utilize emergency services at higher rates than the insured and utilize routine office-based services at lower rates than the insured, which is consistent with the literature [1, 2]. Uninsured households face significant non-price rationing related barriers that limit their ability to access routine services, leading this group to seek care in the emergency room setting [16]. Unfortunately, emergency rooms are among the most expensive places to seek out care and represent a significant uncompensated care cost to both hospitals and state agencies. Second, privately insured patients have the highest propensity to consume routine office-based visits, while publicly insured patients have the highest propensity to consume a mixture of routine and emergency care. Similar to the uninsured group, the increased propensity of utilizing a mixture of routine and emergency care is likely due to non-price rationing factors that make care accessed through a hospital emergency department more accessible than care sought in the routine office-based setting [17]. When health insurance is decomposed into specific public and private insurance types to control for variations in copayments, deductibles, and gatekeeping, the subgroup analysis demonstrates that the main effects are being driven largely by private group insurance and publicly financed Medicaid insurance.

When modeling patient demand for medical care, studies typically focus their analysis on the effects of individual attributes of health insurance on aggregate measures of healthcare utilization [1, 3, 4, 18, 19]. There are two drawbacks to this approach regarding understanding how coverage status directly influences health disparities and inequities. First, studies that focus on copayment rates or provider generosity typically focus on understanding how these factors impact service intensity and quality. However, modeling differences in service intensity alone will not explain low-value patterns of care use [17]. Second, with an exception to emergency situations, an individual’s consumption of medical care is voluntary and driven by price and non-price barriers. Therefore, analysis focused on extensive or intensive margin use of individual services will not reveal patterns of medical services demanded.

There are four main limitations to this paper. First, this study uses repeated cross-section data, which means the evolution of utilization behavior over time cannot be examined. However, this study does examine the timing of diagnosis (Table 5, Panel A) and these results are constant with the main analysis presented in the results. Second, emergency room use is assumed to be voluntary, but it may be the case that patients have no control over their use of the emergency room. This concern was addressed in the design of the medical treatment bundles, where singleton visits only are treated as one or less visits, while the exclusive use of the emergency room to manage their healthcare needs captures a pattern of voluntary use to use the emergency room over other treatments. Third, due to a lack of comprehensive information on disease status and severity, a global disease indicator could not be included as a covariate to control for disease status directly. To overcome this challenge, a subgroup analysis was conducted to include four major chronic diseases: hypertension, coronary heart disease and stroke, diabetes, and cancer. Last, the main results of this study are observational and do not exploit any sources of exogenous variation to explain transitions across insurance states. However, models in this study that use the exogenous variation in the timing of acute health events and an age-based eligibility threshold for Medicare produce results consistent with the main results.

## Conclusions

This paper shows that the insured are more likely to use appropriate levels of medical services than the uninsured. Among the insured, privately insured patients report the highest propensity of using cost-effective routine medical care while the publicly insured report the highest propensity to use the least cost-effective mixture of medical services. The analysis contained within this paper demonstrates that these trends not only exist for the overall population, but also across racial/ethnic, major chronic disease subgroup analyses, and alternative/quasi-experiment models. These findings have important implications for the surveillance of inequities in health, understanding the determinants of medical cost growth, the design of utilization incentives for integrated care, and the structure future health insurance coverage expansions should take to generate individual and system-wide cost savings.

## Data Availability

This study uses public-use data from the Medical Expenditure Panel Survey. All data can be found at: https://www.meps.ahrq.gov/mepsweb/index.jsp

## Appendix A

### Theoretical Framework

Patient demand for appropriate medical care is summarized within a random utility model framework, where individuals are assumed to be utility maximizers [8–10, 15]. There are three types of utility maximizing individuals: an individual with public insurance coverage, an individual with private insurance coverage, and an individual with no insurance coverage. Each type of individual, indexed by *i*, faces a discrete choice decision between *J* treatment options indexed from *j* ∈ {*OB*, *ER*, *MX*}: whether to make routine office-based visits only (*OB*), emergency room visits only (*ER*), or any mixture of routine office-based visits and emergency room visits (*MX*).

Each purchase occasion, an individual uses their budgeted income for medical care ${y}_i^m$ to select a treatment option *j* that maximizes their utility. The treatment decision is modeled as follows:

1 $$ \underset{j,z}{\max }{U}_i\left(H,z\right)\ s.t.{p}_j^i+{p}_zz={y}_i^m\  and\ {H}_i={H}_i\left({x}_j,{\theta}_i\right) $$

where *θ*_*i*_ represents pre-treatment health state with larger values indicating worse state of health, *x*_*j*_ and characters of treatment bundle *j*, ${p}_j^i$ is the price of treatment bundle *j* that individual *i* faces using their insurance, *z* is an outside option, and *p*_*z*_ is the price of the outside option *z*. *H*_*i*_(∙) and *H*_*i*_ are the health production function and post-treatment health for individual *i*, respectively.

As *z* (denoted *j* = 0) is the non-purchase of any treatment in *J*, *z* can be viewed as a fourth treatment option consisting of no medical visits. Alternatively, *z* can be viewed as some other form of medical investment used to maximize utility. The model assumes *H*_*i*_(∙) is differentiable and that the following initial condition holds:

2 $$ {H}_i\left(0,{\theta}_i\right)=-\theta $$

This means that if the patient *i* does not consume a treatment bundle in *J*, then their post-treatment health state is equal to their pre-treatment health state [15].

Substituting the budget constraint into Eq. (1), the patient’s problem is now selecting the treatment bundle that gives them the highest conditional indirect utility:

3 $$ \underset{j}{\max }{U}_j\left({H}_{ij}\left({x}_{ij},\theta \right),{p}_j,{p}_z,{y}_i^m\right)={V}_{ij}\left({H}_{ij}\left({x}_{ij},\theta \right),{p}_j,{p}_z,{y}_i^m\right)+{\epsilon}_{ij} $$

where *V*_*ij*_(∙) is the indirect utility from all of the observable characteristics and *ϵ*_*ij*_ is the unobserved utility that equates *V*_*ij*_(∙) to the actual utility of reach individual. An individual treatment bundle is selected if and only if a patient selects a treatment bundle *j* over all alternative treatment bundles *k* ∈ *J and k* ≠ *j*:

4 $$ {V}_{ij}\left(\bullet \right)>{V}_{ik}\left(\bullet \right)\forall k\ne j $$

### Empirical Specification

Following the discrete choice model of demand for medical treatment derived in Appendix A, Section 1, a multinomial logistic regression (MLR) model is used to estimate the probability of selecting an alternative medical treatment bundle based on health insurance type. The probability that an individual *i* will choose a medical treatment bundle *j* over *j*′ lies between 0 and 1. The model assumes that individuals select the medical treatment bundle that maximizes their utility [10]. If a linear structure is imposed on the conditional indirect utility function of *i* (Eq. 3), the model can be expressed as follows:

5 $$ \mathit{\Pr}\left[{Y}_i=j\right]=\frac{\exp \left({\beta}_j{X}_i\right)}{\sum_{j=0}^J\exp \left({\beta}_j{X}_i\right)} $$

where *Pr*[*Y*_*i*_ = *j*] is the probability of choosing either routine visits only, emergency room visits only, or any mixture of routine and emergency room visits, with no medical visits as the reference category. *J* is the number of treatments in the choice set, *j* = 0 is no medical visits, and *β*_*j*_ and *X*_*i*_ are a vector of estimated parameters and independent controls, respectively. If the logit equation above (Eq. 4) is rearranged and exponentiated, the multinomial logistic regression equation used to estimate the coefficients is as follows:

6 $$ \ln \left[\frac{P_i}{1-{P}_i}\right]={b}_0+{b}_1{x}_1+\dots +{b}_v{x}_v $$

From the previous equation (Eq. 5), where *ln*[*P*_*i*_/(1 − *P*_*i*_ )], the log odds ratio, is a linear function of independent controls, *X*_*i*_. In this paper, the following independent controls are considered: health insurance coverage status, geographic region, race and ethnicity, age in years, gender, educational attainment, and total out-of-pocket expenditures on medical care.

## References

[1] Anderson M, Dobkin C, Gross T. The effect of health insurance coverage on the use of medical services. Am Econ J Econ Policy. 2012;4(1):1–27. https://www.aeaweb.org/articles?id=10.1257/pol.4.1.1.

[2] Ayanian JZ, Weissman JS, Schneider EC, Ginsburg JA, Zaslavsky AM (2000). Unmet health needs of uninsured adults in the United States. JAMA.

[3] Card D, Dobkin C, Maestas N (2008). The impact of nearly universal insurance coverage on health care utilization: evidence from Medicare. Am Econ Rev.

[4] Card D, Dobkin C, Maestas N (2009). DOES MEDICARE SAVE LIVES?. Q J Econ.

[5] Currie J, Gruber J (1996). Health insurance eligibility, utilization of medical care, and child health. Q J Econ.

[6] Kronick R (2009). Health insurance coverage and mortality revisited. Health Serv Res.

[7] Lillie-Blanton M, Hoffman C (2005). The role of health insurance coverage in reducing racial/ethnic disparities in health care. Health Aff.

[8] Aldrich JH, Nelson FD. Quantitative applications in the social sciences: linear probability, logit, and probit models. Thousand Oaks: SAGE Publications, Inc.; 1984. 10.4135/9781412984744, https://methods.sagepub.com/book/linear-probability-logit-and-probit-models.

[9] Gertler P, Locay L, Sanderson W (1987). Are user fees regressive?: the welfare implications of health care financing proposals in Peru. J Econ.

[10] McFadden DL, Zarembka P (1973). Conditional Logit Analysis of Qualitative Choice Behavior. Frontiers in Econometrics.

[11] McFadden DL, Hildenbrand W (1983). Qualitative Response Models. Advances in Econometrics.

[12] D’Agostino RB, Vasan RS, Pencina MJ, Wolf PA, Cobain M, Massaro JM, Kannel WB (2008). General cardiovascular risk profile for use in primary care: the Framingham heart study. Circulation.

[13] Dugan J, Virani SS, Ho V (2012). Medicare eligibility and physician utilization among adults with coronary heart disease and stroke. Med Care.

[14] Kim AS, Claiborne Johnston S (2011). Global variation in the relative burden of stroke and ischemic heart disease. Circulation.

[15] Brand, Keith. 2005. “A Structural Model of Health Plan Choice and Health Care Demand in the Medicare Managed Care Program.” *Unpublished Ph. D. Dissertation, University of Virginia.[86]*.

[16] Reinhardt UE (1996). Rationing health care: what it is, what it is not, and why cannot avoid it. Baxter Health Policy Rev.

[17] Kangovi S, Barg FK, Carter T, Long JA, Shannon R, Grande D (2013). Understanding why patients of low socioeconomic status prefer hospitals over ambulatory care. Health Aff.

[18] Aron-Dine A, Einav L, Finkelstein A (2013). The RAND health insurance experiment, three decades later. J Econ Perspect.

[19] Dor A, Farley DE (1996). Payment source and the cost of hospital care: evidence from a multiproduct cost function with multiple payers. J Health Econ.

